# One-step peracetic acid pretreatment of hardwood and softwood biomass for platform chemicals production

**DOI:** 10.1038/s41598-021-90667-9

**Published:** 2021-05-27

**Authors:** Chandan Kundu, Shanthi Priya Samudrala, Mahmud Arman Kibria, Sankar Bhattacharya

**Affiliations:** Department of Chemical Engineering, Monash University, Melbourne, 3800 Australia

**Keywords:** Chemical engineering, Solid biofuels

## Abstract

Lignocellulosic biomass is an attractive renewable resource to produce biofuel or platform chemicals. Efficient and cost-effective conversion systems of lignocellulosic biomass depend on their appropriate pretreatment processes. Alkali or dilute acid pretreatment of biomass requires a high temperature (> 150 °C) to remove xylan (hemicellulosic sugar) and lignin partially. In this study, peracetic acid was used to pretreat biomass feedstocks, including hardwood and softwood species. It was found that the thermally-assisted dilute acid pretreatment of biomass conducted under the mild temperature of 90 °C up to 5 h resulted in the effective removal of lignin from the biomass with a negligible loss of carbohydrates. This thermally-assisted pretreatment achieved 90% of delignification, and this result was compared with the microwave-assisted pretreatment method. In addition, the crystallinity index (*CrI*), surface morphology, and chemical structure were significantly changed after the acid pretreatment. The biomass digestibility increased significantly with increased reaction time, by 32% and 23% for hardwood and softwood, respectively. From this study, it is clear that peracetic acid pretreatment is an effective method to enrich glucan content in biomass by delignification.

## Introduction

The ever-increasing global energy demand has so far been based on the use of fossil fuels, which has several adverse effects, such as the release of carbon dioxide, a significant contributor to climate change. At the same time, the ever-increasing energy demand calls for the exploration of alternative sources of renewable energy like solar thermal, wind, geothermal, hydrothermal, and biomass. In the last few decades, biomass has attracted significant attention as a renewable resource due to its high fixed carbon content, abundance in nature, low cost, and environmental-friendliness^1,2^. Moreover, biomass can be used to sequestrate atmospheric carbon dioxide through carbon fixation^3^. These advantages have prompted the chemical industries to explore the utilization of biomass as an alternative feedstock for bio-oil as well as chemical synthesis. Lignocellulosic biomass can be converted to bio-oil and chemicals through thermochemical and biochemical processes. Thermochemical methods are fast and can potentially be cost-effective, whereas biochemical processes are time-consuming due to microorganisms and enzymes usage.

Lignocellulosic biomass consists predominantly of three polymers, namely cellulose, hemicellulose, and lignin. Cellulose is the major structural component that acts as a natural scaffold in the form of cellulose microfibrils. Cellulose is difficult to degrade due to the complex structure of biomass and the high stability of its microfibers, which makes bio-oil production from such biomass a real challenge. In fact, a low conversion efficiency coupled with high energy consumption is the primary barrier to the commercialization of bio-oil production from lignocellulosic biomass^4^. However, a plausible way of maximizing bio-oil yield is to control the thermochemical reactions underlying fast pyrolysis of biomass to ensure cellulose depolymerization and preventing it from further cracking. But, the other component of biomass, lignin, may make the process difficult. Hence, effective delignification before the thermochemical process is advantageous, which can be achieved through biomass pretreatment that enhances both the yield and the desired products' selectivity.

The effectiveness of pretreatment can be enhanced either by removing hemicellulose or delignification. Pretreatment is required to break the interaction of lignin seal with hemicellulose and disrupt the cellulose's crystalline structure. These pretreatment strategies include the use of mineral or organic acid, alkali, ionic liquid, steam explosion, or hydrothermal treatment^5^.

Dilute acid pretreatment at high temperatures (> 150 °C) is known to be the most effective process for removing hemicellulose from biomass. Since lignin provides a negative effect on cellulose conversion, pretreatment's success relies on the maximum delignification with minimal glucan (a main component of cellulose) loss^6^. Delignification from a strong cell wall of biomass is a challenge while ensuring minimal damage to cellulose. Ammonia fiber explosion (AFEX), organosolv, and alkali pretreatment can partially breakdown the lignin and hemicellulose but in that context, the literature has so far focused on enzymatic hydrolysis of biomass^6,7^ and bioethanol production^8^. Another research indicated that peracetic acid pretreatment could effectively remove 90% of lignin from a hardwood species yellow poplar with a commensurate increase in the enzymatic digestibility^6,9^. Peracetic acid is a strong oxidant that can be prepared by mixing hydrogen peroxide with acetic acid in the presence of a sulphuric acid catalyst. This peracetic acid is an effective pretreatment method to delignify biomass by leaving minimum lignin, which maximizes the chance of converting the cellulosic portion^10,11^. Also, selective delignification of biomass enhances the surface area, pore size, and porosity of the cell wall, maximizing cellulose conversion during saccharification and hydrolysis process^8^.

Fewer studies have addressed the different reactions occurring during the thermochemical conversion of biomass and cellulose for chemicals production. A method suggested that phosphoric acid pretreatment of pine biomass followed by injection of water to the pyrolysis vapor increased levoglucosenone production by 30.7%^12,13^. Another study showed the catalytic effect of sulphuric and hydrochloric acid could boost levoglucosenone, levulinic acid, and furans production during thermochemical reaction^14,15^. A few other studies described the composition and functional group changes of the biomass during the thermochemical process but not for the delignified feedstock^16,17^. Nonetheless, there is a dearth of detailed studies on delignification mediated thermochemical conversion of biomass to platform chemicals.

As a source of woody biomass, victorian ash which is a hardwood species and pine which is a softwood species have been used in this study. These species are naturally grown and abundant in Australia. The biomass are collected from a timber plant in Australia and contain the waste remnants after timber processing with current use. The 2007 data from the Australian Bureau of Statistics points to a figure of around 1,781,000 tonnes of wood waste generated in Australia per annum^18^. The main origins of hardwood and softwood wastes were construction, timber processing industry, and demolition waste^19^.

This study is aimed at investigating convenient ways to increase the delignification of hardwood and softwood biomass in a low reaction temperature. Finding a proper pretreatment process that will remove most of the lignin while preserving the maximum carbohydrates is a prerequisite objective for this investigation. This study is part of a wider study where we have demonstrated that delignified biomass can be more effectively used to produce high-value platform chemicals—levoglucosenone and 5-Chloromethyl furfuryl. In this study, two types of pretreatment, namely thermally-assisted and microwave-assisted, are studied in peracetic acid environment to maximize the delignification with minimal carbohydrate degradation. The pretreated biomass has been characterized by compositional and morphological analyses.

## Results and discussion

### Changes in biomass yield and chemical composition

One of the main focus of our study was to investigate the effect of pretreatment time on the solid recovery (%) of biomass as well as chemical composition, thereof. This was accomplished at a constant temperature of 90 °C and the data has been summarized in Table 1 that lead to a few significant and interesting inferences.

**Table 1:** Biomass composition of untreated and acid pretreated hardwood and softwood biomass during thermally-assisted pretreatment.

Pretreatment (time)	Glucan (%)	Xylan and/or mannan (%)	Arabinan (%)	Galactan %	AIL (%)^a^	ASL (%)	Lignin (%)	Solid recovery (%)
Raw Hardwood	40.51 (3.15)	22.12 (1.62)	1.06 (0.05)	ND	29.65 (1.64)	2.59 (0.09)	32.24	
Hardwood 30 min	59.57 (1.08)	18.22 (0.69)	1.68 (0.01)	ND	11.67 (0.50)	2.19 (0.22)	13.86	62.08
Hardwood 1 h	66.30 (1.87)	16.09 (0.37)	1.70 (0.06)	ND	9.53 (0.75)	2.26 (0.21)	11.79	55.07
Hardwood 2 h	67.69 (0.91)	14.09 (0.25)	1.63 (0.04)	ND	7.58 (0.67)	1.16 (0.10)	8.74	53.93
Hardwood 3 h	69.53 (2.63)	13.37 (0.59)	1.61 (0.03)	ND	5.98 (0.49)	1.19 (0.05)	7.17	53.32
Hardwood 4 h	72.14 (0.39)	12.18 (0.40)	1.53 (0.06)	ND	5.01 (0.24)	1.14 (0.07)	6.15	51.66
Hardwood 5 h	73.78 (1.30)	11.40 (0.37)	1.39 (0.07)	ND	4.80 (0.54)	1.05 (0.07)	5.85	48.60
Raw Softwood	39.62 (0.49)	21.77 (1.13)	1.31 (0.01)	0.36 (0.03)	34.27 (0.35)	2.43 (0.05)	36.70	
Softwood 30 min	47.24 (0.77)	20.44 (1.05)	1.09 (0.02)	0.32 (0.01)	22.86 (0.26)	2.19 (0.03)	25.05	80.48
Softwood 1 h	49.32 (1.93)	19.9 (0.73)	1.12 (0.06)	0.35 (0.02)	19.49 (0.53)	2.33 (0.23)	21.81	78.38
Softwood 2 h	60.53 (1.70)	18.33 (1.52)	1.06 (0.13)	0.27 (0.01)	13.90 (1.30)	2.13 (0.25)	16.03	61.19
Softwood 3 h	65.19 (2.60)	15.42 (0.64)	0.52 (0.01)	0.22 (0.09)	12.65 (0.21)	2.28 (0.18)	14.93	58.43
Softwood 4 h	67.51 (0.90)	13.45 (0.64)	0.52 (0.06)	0.29 (0.01)	11.46 (1.21)	2.22 (0.18)	13.67	56.26
Softwood 5 h	70.83 (0.56)	11.78 (0.55)	0.40 (0.03)	0.21 (0.03)	8.37 (1.62)	2.27 (0.14)	10.64	53.74

Here, Lignin % = acid insoluble lignin (AIL %) + acid soluble lignin (ASL %) and *ND* not detected. The parentheses contain the standard deviation with the analysis repeated thrice.

Firstly, most of the weight loss resulting from thermally-assisted acid pretreatment can be attributed to lignin removal with only partial contributions from hemicellulosic sugar degradation for both the biomass species studied here. Secondly, there was a steady decrease in % solid recovery with increasing pretreatment time is envisaged for both hardwood and softwood that is a clear indication of enhanced lignin removal as a function of pretreatment residence time. This trend was found for the pretreatment of both agriculture residue and wood biomass in the presence of hydrogen peroxide (H_2_O_2_)-acetic acid (CH_3_COOH) (HPAC)^11,20^. However, the digestion of biomass during hardwood pretreatment was much faster than softwood pretreatment since 1-h pretreatment could recover only 55.07% of hardwood biomass as compared to 73.38% for softwood. This comparatively lower extent of delignification can be attributed to the complex structure of hemicellulose (a galactoglucomannan backbone with slightly branched chains (1 → 4) linked with β-d-glucopyranose and β-d-mannopyranose units) in softwood, which is hard to degrade^21^. Also, the total lignin content in hardwood is lower than the softwood, which plays a vital role in removing the lignin from the complex biomass structure^22^.

Compared to raw biomass, the glucan content of pretreated biomass increased remarkably as a result of delignification. For instance, after 1-h of pretreatment, glucan content increased by about 26% and 10% for hardwood and softwood respectively (Table 1). However, the increasing reaction time after 3-h only had a minimal impact on delignification, particularly in hardwood. Thus, the glucan recovery and delignification showed the 5-h peracetic acid pretreatment is the best condition.

Although the microwave-assisted pretreatment method is widely used in laboratories^23,24^ for its high efficiency in hemicellulose and lignin removal^6^, hardwood pretreatment showed a lesser delignification (higher solid recovery) with this method (Supplementary Tables S1 and S2) as compared to the thermally-assisted pretreatment technique. Such low lignin removal was an effect of lower reaction temperature and short reaction time compared to the thermally-assisted pretreatment discussed previously. Similar trends have been observed with softwood microwave-assisted pretreatment (30-min and 1-h) as well. Nonetheless, uniform heating and selective processing are inherent advantages of microwave-assisted pretreatment. Also, this microwave-assisted pretreatment method is a good alternative for reducing the pretreatment time and hydrolysis^25^. Thus, at low temperature (for example, at 90 °C), microwave-assisted pretreatment needs to operate for a longer time span to achieve as effective a delignification as is obtained by thermally-assisted pretreatment.

### Recovery of carbohydrate after pretreatment

**Figure 1:**
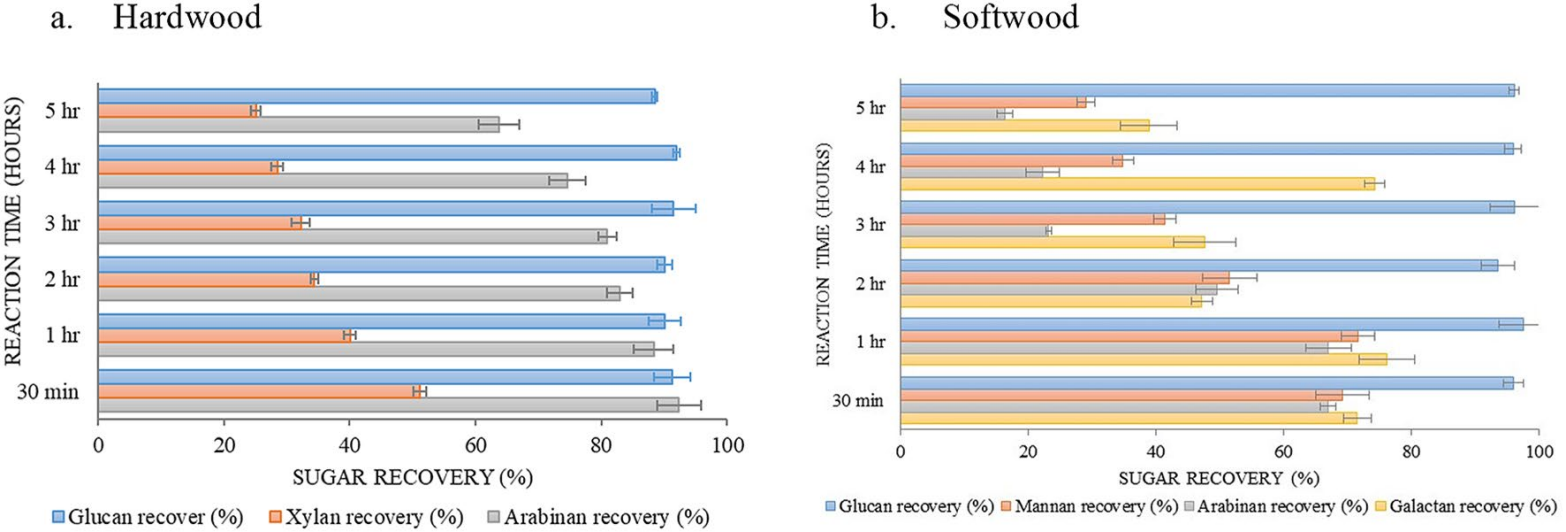
Carbohydrate recovery rates after the acid pretreatment.

The rates of carbohydrate recovery (expressed as glucan, xylan, and arabinan for the hardwood; glucan, mannan, arabinan, and galactan for the softwood) at different reaction times of thermally-assisted pretreatment method are presented in Fig. 1a,b. The recovery of cellulosic sugar (glucan) showed a consistent result with a maximum of 10% total loss. Also, it is noted that the recovery of other carbohydrates (xylan, mannan, arabinan, galactan) is much lower than that of glucan. We observed a steady reduction in the recovery of xylan in hardwood and mannan in softwood upon increasing the pretreatment duration (Fig. 1a,b). This clearly indicates that increasing the time span of acid treatment accelerated the removal of easily degradable hemicellulose rather than the more stable cellulosic part of biomass^5^. Himmel et al. suggested that the full or partial removal of hemicellulose helps to expose the crystalline cellulose core of cell-wall microfibrils, and it may increase the cellulose degradation during conversion through hydrolysis^26^.

The rates of carbohydrate recovery with microwave-assisted pretreatment methods for both hardwood and softwood are presented in Supplementary Tables S1 and S2, respectively. Both the species showed 90–95% of glucan recovery after pretreatment. With an increase in reaction time, the recovery of all sugars except glucan decreased. A similar trend has been reported during the delignification of poplar biomass^8^. Thus, the digestibility of lignin and other sugars did not affect much on glucan recovery even in both pretreatment method.

### Delignification rate of pretreated biomass

Table 2 describes the rate of delignification for different reaction times used in the thermally-assisted pretreatment method. The lignin recovery represents delignification efficiency. Only 30 min of thermally-assisted pretreatment removed about 75% of hardwood and 46% of softwood lignin. After 5 h of pretreatment, the acid-insoluble lignin recovery of hardwood resulted in a delignification of 92.13% as depicted by compositional analysis. Similarly, 5-h pretreatment of softwood showed 86.88% delignification (Table 2). Thus the lower recovery of solid at longer reaction times is related to the removal of lignin.

**Table 2:** Delignification rate (%) of untreated and pretreated biomass of hardwood and softwood.

Thermally-assisted pretreatment			Microwave-assisted pretreatment		
(Time-h)	Hardwood	Softwood	(Time-min)	Hardwood	Softwood
30 min	75.57 (1.05)	46.33 (0.60)	10 min	51.05 (2.98)	26.27 (2.23)
1 h	82.30 (1.39)	55.43 (1.22)	20 min	59.83 (1.02)	34.94 (2.79)
2 h	86.22 (1.22)	75.18 (2.31)	30 min	64.89 (2.75)	44.06 (2.63)
3 h	89.24 (0.87)	78.43 (0.36)	1 h	75.66 (1.46)	51.67 (0.71)
4 h	91.27 (0.42)	81.19 (1.98)			
5 h	92.13 (0.89)	86.88 (2.54)			

The parentheses contain the standard deviation with the analysis repeated thrice.

The rate of delignification is lower with softwood than hardwood, possibly because the structure of softwood is more recalcitrant than hardwood, and the higher lignin content of softwood is always a matter of concern^22^. Due to the structure of softwood lignin (predominantly guaiacyl lignin), 70% of which is located in the secondary walls, and are polymerization products of coniferyl alcohol. By contrast, hardwood lignin is typically copolymer of coniferyl and sinapyl alcohol^21^.

Microwave-assisted pretreatment provided 64.89% and 46.58% of delignification for 30-min of reaction time at 90 °C for hardwood and softwood, respectively. By comparison with 30-min pretreatment, the delignification followed the thermally-assisted pretreatment efficiency for the softwood. However, 30-min hardwood microwave-assisted pretreatment reaction showed less efficacy compared to thermally-assisted pretreatment. Compared to the microwave pretreatment, 1-h thermally-assisted pretreatment resulted in increased delignification by around 7% (Table 2). The microwave-assisted pretreatment is faster and more effective at high temperatures (> 120 or 130 °C). It was reported that alkaline pretreatment assisted with microwave above 140 °C could remove around 40% of lignin from switchgrass. Both cellulosic and hemicellulosic sugar loss increased upon extending the reaction time^27^. In contrast, this study showed the delignification with a minimal cellulosic (glucan) loss at a lower temperature.

### Mass change of components after the pretreatment

Raw hardwood biomass contains glucan 40.51 g, xylan 22.12 g, arabinan 1.06 g and 33.53 g of lignin. Peracetic acid pretreatment selectively hydrolyzed and solubilized lignin and xylan, resulting in increased relative amounts of glucan in the remaining solid. Understanding the component change and carbohydrate degradation is essential to achieve high cellulose yield along with extensive delignification^28^. During 1-h of pretreatment, about 4 g of glucan, 16 g of xylan, and 25 g of lignin were digested in the hydrolysate. By increasing the reaction time, xylan and lignin were dissolved in the hydrolysate, and the result supported the hardwood delignification^29^. The glucan content remained the same until the 5-h of pretreatment but about 75% of xylan and 90% of lignin were removed from the solid biomass (Supplementary Fig. S1a). Literature suggested that at higher temperature, acid pretreatment can effectively remove 80–90% of xylan^30^ but at the mild condition of 90 °C acid treatment removed 60–75% of the xylan. However, this result showed most of the glucan were present in the solid portion of the biomass. Another literature showed around 90% of lignin removal efficiency of yellow poplar which is a hardwood species at 120 °C^6^, and this study showed more than 90% of lignin removed thorough the thermally-assisted process without using any precise instrument like the microwave.

100 g of raw softwood biomass contains 39.62 g glucan, 21.77 g mannan, 1.31 g galactan, and 36.70 g lignin. Xylan and mannan hydrolyzed from the hemicellulose of the softwood which is constructed by galactoglucomannans and arabinoglucuronoxylan^21,29^. Unlike hardwood, the glucan content remained almost the same in the softwood after different reaction times of pretreatment. With the increase in pretreatment time, mannan and lignin concentration decreased significantly. More than half of the mannan was removed after 3 h of pretreatment, and only 6.73 g of lignin was in the solid (Supplementary Fig. S1b).

### Characterization of biomass

#### XRD analysis

**Figure 2:**
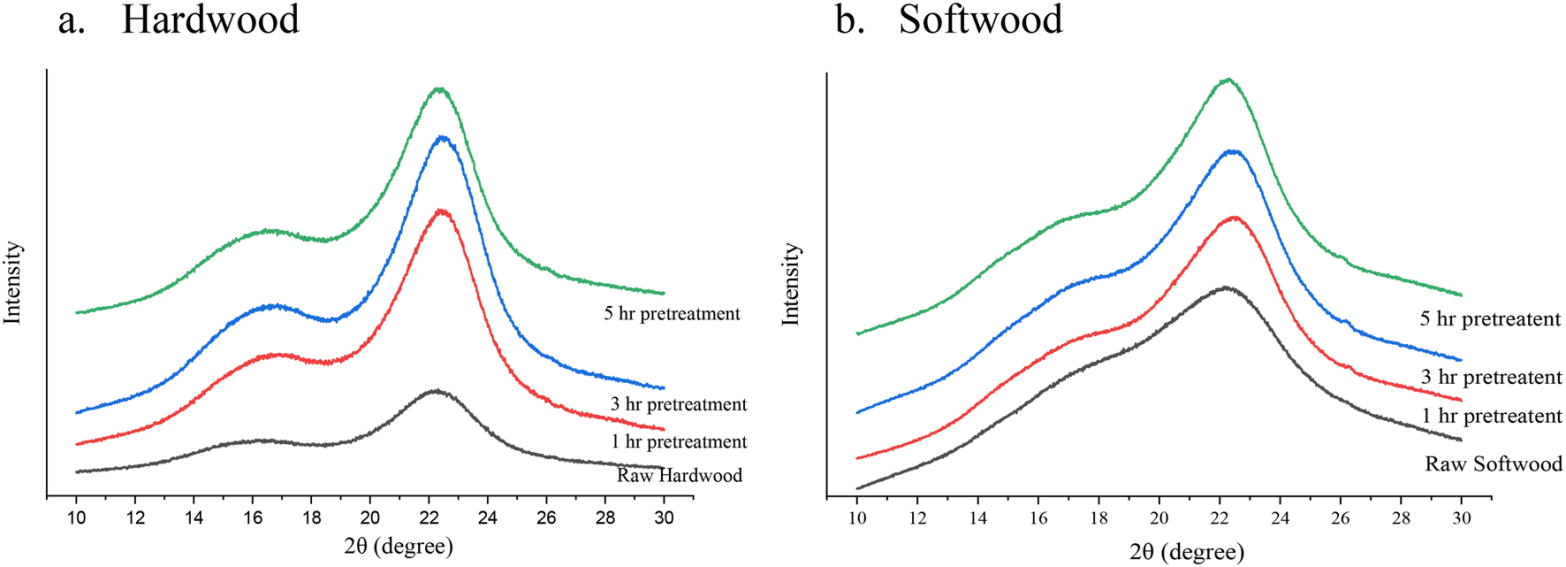
Crystallinity changes between untreated and pretreated biomass.

Figure 2a describes the XRD intensity for hardwood biomass. As can be seen, raw hardwood had a decrease in peak intensity compared to pretreated biomass. This matches well with the crystallinity index (*CrI*) reported in method section. According to our calculations, the *CrI* of raw hardwood is 54.20, which increased to 56.36 after 1-h pretreatment due to the removal of lignin and the amorphous xylan fraction. The *CrI* further increased to 60.15 for 3 h of pretreatment, but then became stagnant at 60.08 for 5 h of pretreatment. This is due to insignificant difference of hemicellulose and lignin removal between 3 and 5 h of pretreatment. In the pretreated biomass two sharp peaks at angles 16° and 22° correspond to lattice planes of crystalline cellulose I polymorph^31,32^. The sharp peaks of pretreated biomass and *CrI* reveal the relative amount of crystalline cellulose in the solid.

Figure 2b describes the XRD intensity of softwood biomass. As can be seen, the *CrI* of raw softwood is 29.11, which is much lower than that of raw hardwood. The chemical structure of the lignin and amorphous portion of hemicellulose can play an important role on *CrI*^33^. A higher digestibility during the pretreatment is obtainable with more crystallinity. Resulting in the *CrI* increased to 40.55 after 1-h of pretreatment. However, the 3-h of reaction time could not effectively change the *CrI* (40.38) can be the same reason that we found in pretreated hardwood. But, the *CrI* slightly changed to 42.08 after 5-h pretreatment can be described as the change of structure from FTIR analysis.

#### Surface morphology of biomass

**Figure 3:**
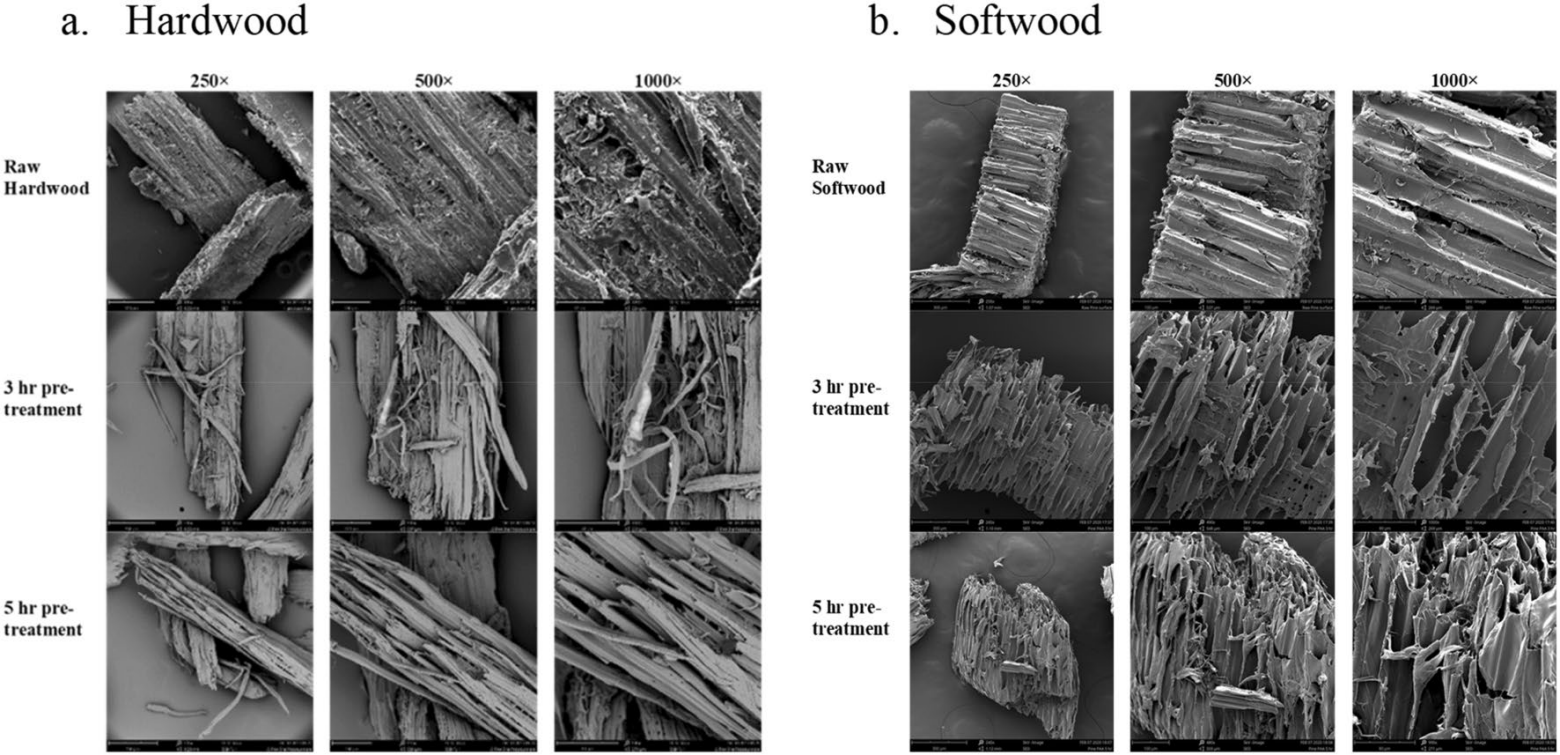
Surface morphology of untreated and acid pretreated biomass.

SEM is a powerful tool widely used to understand the surface morphology of lignocelluloses^33^. Untreated hardwood showed a compact and rigid surface with lignin droplets while pretreated hardwood exhibited a corrugated and rough surface (Fig. 3a). The surface morphology of pretreated hardwood had a totally different structure. The more porous and disrupted topography with an appearance of softness was observed in pretreated biomass samples, especially in biomass treated for 5-h. This change of morphology revealed the disruption of tissue network of the biomass and exposed cellulose like fibers resulting in more glucan content in pretreated biomass.

Untreated softwood and pretreated biomass showed similar types of surface morphology as hardwood. The surface of untreated softwood covered with lignin droplets. 3- and 5-h pretreated softwood showed enlarged pores, fragmented structure, and irregular cracks (Fig. 3b). Similar kind of results has been found in the pretreatment of sugarcane bagasse^34^. Although, after 5-h of acid pretreatment, a few pores were found in hardwood biomass, in softwood, most of the pores had disappeared, and a smooth surface appeared due to the removal of lignin.

#### Fourier transform infra-red spectroscopy (FTIR) of biomass

**Figure 4:**
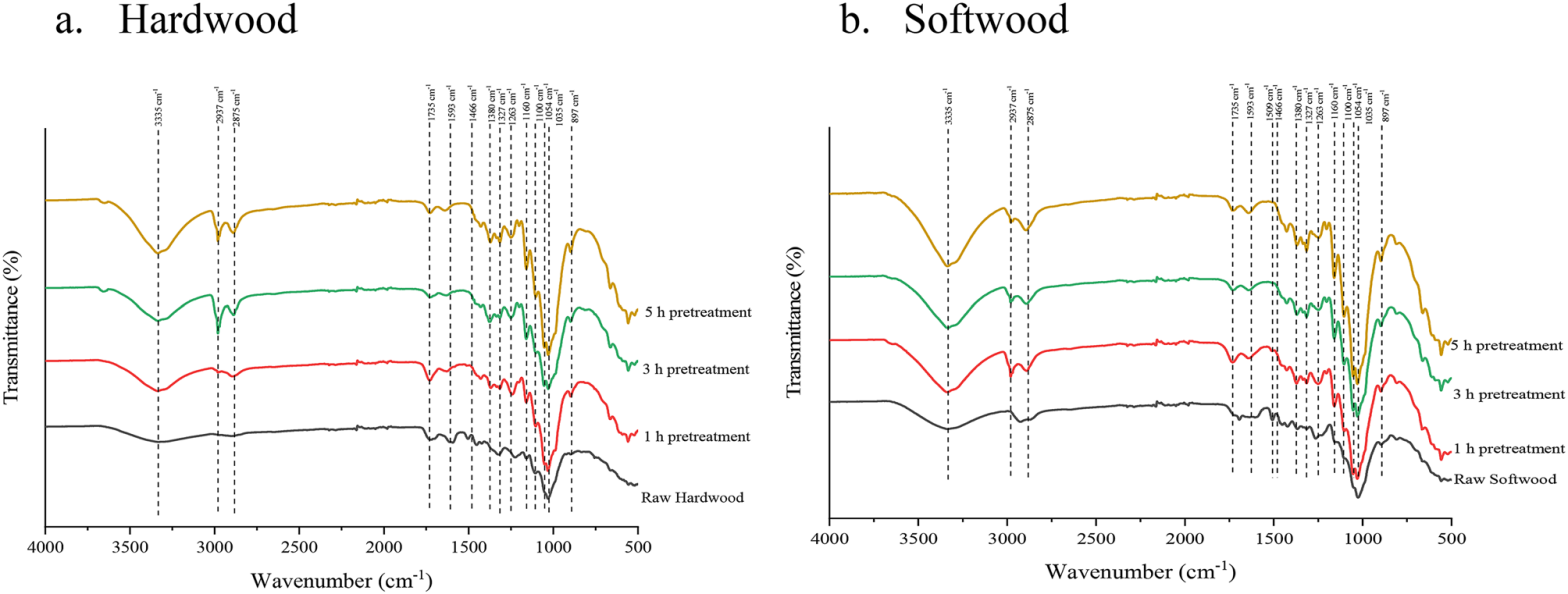
FTIR spectra of untreated and pretreated biomass.

Figure 4a,b present the FTIR spectra of untreated and pretreated hardwood and softwood respectively in different reaction times. Compared to the raw hardwood, pretreated biomass showed significant change main functional group 3335 cm^−1^ associated with the increase of cellulose concentration and the range of 3000–2800 cm^−1^ region due to delignification. The new notable bands appear between 3000 and 2800 cm^−1^ due to the pretreatment effect on the methyl and methylene group of lignin by CH_2_ and CH_3_ stretching^35^. The peak at 1735 cm^−1^ represents carbonyl stretching of unconjugated ketones. The peak shape becomes narrower in the pretreated hardwood biomass due to C=O stretching vibration in the acetyl group of hemicellulose. This deformation at 1735 cm^−1^ band is indicating the hemicellulose dissolution during the pretreatment. The transmittance band 1593 cm^−1^ related to aromatic vibration of C=O stretching almost disappeared in the pretreated sample due to delignification.

Furthermore, the band at 1466 cm^−1^ related to the C-H deformation of lignin and hemicellulose can be seen in the pretreated biomass. The band at 1160 cm^−1^ represents the C–O–C glycosidic bond, 1100 cm^−1^ represents C–O–C ring skeleton vibration, and 1035 cm^−1^ represents C–O–H stretching of alcohol, which are intense in raw hardwood. With the reaction time, the pretreated biomass showed a consistent increase in the relative amount of glucan.

As can be seen in Fig. 4a,b, at 3 and 5-h pretreatment the transmittance band related to hemicellulose and lignin decreased significantly compared to 1 h pretreatment. This difference proved that the solid remained after pretreatment of 3 and 5-h, leaving a glucan-enriched solid and with trivial lignin content.

**Figure 5:**
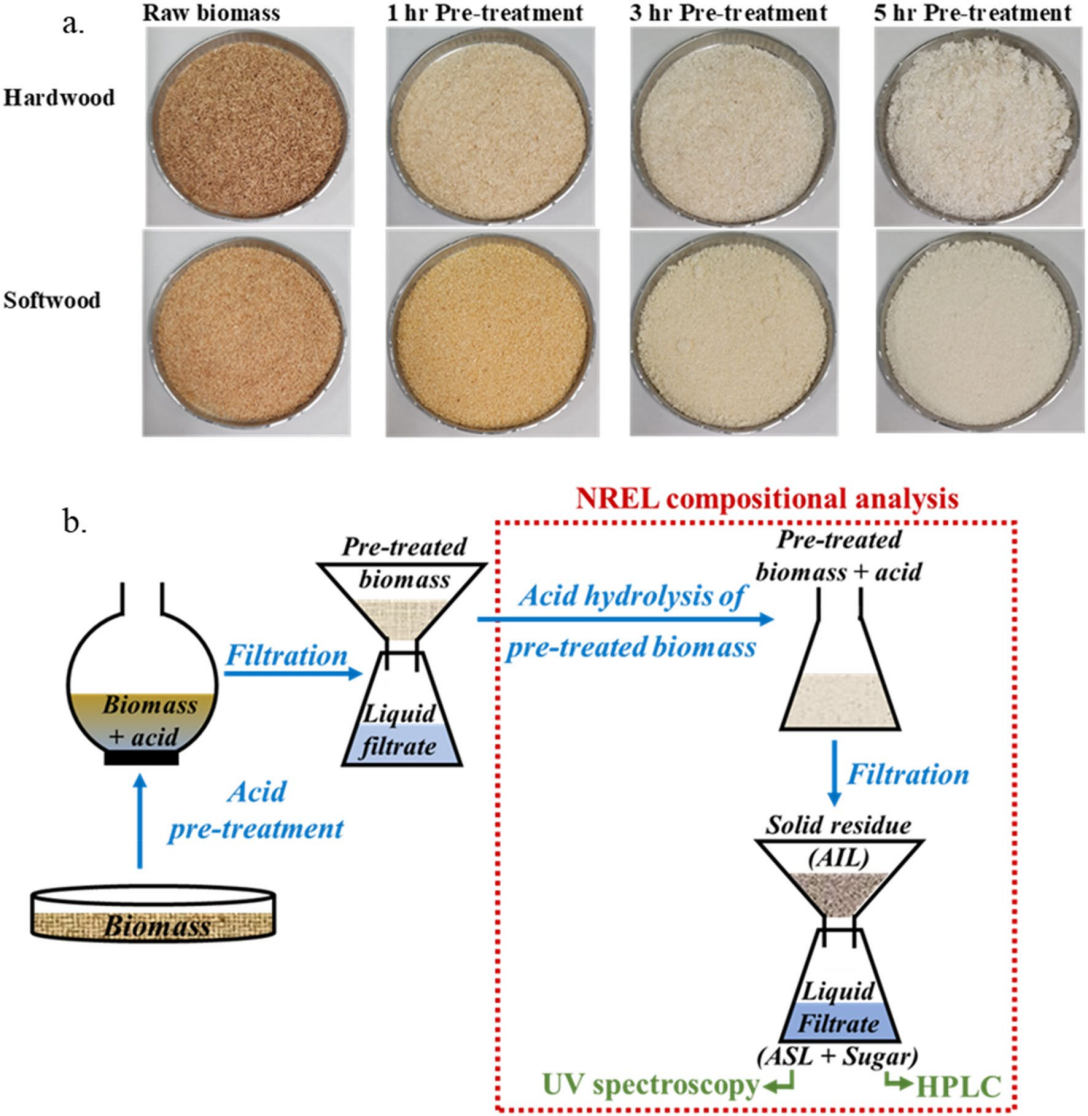
Color difference and physical appearance of untreated and acid pretreated biomass thermally-assisted pretreatment method using peracetic acid. Lower diagram shows a schematic of biomass pretreatment and NREL compositional analysis of the untreated and pretreated samples.

Figure 5 represents the FTIR spectra of untreated and pretreated softwood with different reaction times. The O–H stretching at 3335 cm^−1^ is associated with the increase of cellulose concentration, and the sharp changes were significant with the extended reaction time of pretreatment. The appearance and disappearance at the transmittance band 2937 cm^−1^ are related to the asymmetric C–H stretching of the lignin part of the biomass due to the chemical changes by delignification. There was an apparent change in the intensity of the transmittance band 2875 cm^−1^ compared to the raw softwood, indicating the loss of carboxyl and methylene group^36^. The peak changes are related to the C–H stretch of cellulose, and the 2875 cm^−1^ band region became narrower that cellulose was becoming stronger with the increased reaction time. A significant change was observed at the transmittance band 1593 cm^−1^ and 1509 cm^−1^ presented the lignin polymer and related to the C=C stretching vibration in the aromatic ring due to the effect of pretreatment^37^. The band at 1509 cm^−1^ and the C–H deformation band at 1466 cm^−1^ completely disappeared due to the lignin removal. Also, the guiacyl ring and C–O stretching vibration represented at 1263 cm^−1^ for all pretreated biomass, and this is consistent with the result obtained by delignification. The bands at 1100 cm^−1^ and 1035 cm^−1^ represents C–O–H which are more intense in 5 h pretreated hardwood provided more crystallinity structure of glucan. Interestingly, the chemical structure and functional group change showed a similar trend between hardwood and softwood biomass.

#### Ultimate analysis

Table 3 describes the C, H, O, N, and ash content of untreated and pretreated biomass for different reaction times. It can be seen that the carbon content is higher in untreated biomass. But, the carbon content of untreated softwood (51.68%) is much higher compared to the untreated hardwood (46.42%), due to the high lignin content in the raw material. As can be observed, both biomasses have a decreasing trend of carbon content properties with the increase of reaction time. In lignocellulosic biomass, the percentage of lignin is proportionally related to the carbon content^38^. The decreasing carbon content resulted in higher digestibility of pretreated biomass is associated with delignification^39^.

**Table 3:** Ultimate analysis (ash-free basis) of untreated and pretreated biomass.

	Nitrogen (%)	Carbon (%)	Hydrogen (%)	Oxygen (%)	Ash (%)
Raw hardwood	0.14	46.42	5.43	48.01	0.67
Hardwood 1 h	0.06	45.62	5.49	48.83	0.38
Hardwood 3 h	0.04	45.49	5.74	48.73	0.32
Hardwood 5 h	0.04	44.72	5.70	49.54	0.29
Raw softwood	0.08	51.68	5.76	42.48	0.36
Softwood 1 h	0.05	46.60	5.71	47.64	0.30
Softwood 3 h	0.04	45.69	5.71	48.56	0.30
Softwood 5 h	0.03	45.35	5.52	49.10	0.29

## Materials and methods

### Feedstock and pretreatment

As a source of woody biomass, victorian ash (produced by two species—*Eucalyptus regnans* and *Eucalyptus delegatensis*, sourced from Victoria, Australia), which is a hardwood species, and pine (*Pinus radiata*) which is a softwood species, have been used in this study. Sawdusts from respective wood samples were air-dried, screened to a nominal size of ~ 500–850 µm, and stored under ambient conditions (1 bar pressure, 25 °C) in an air-tight plastic zipper bag. All of the analyses were carried out on a dry-weight basis.

Pretreatment of the as-prepared biomass sawdust was carried out in the presence of peracetic acid. Peracetic acid was prepared by mixing hydrogen peroxide and acetic acid in a volumetric ratio of 1 to 1 in the presence of 1 wt% sulphuric acid catalyst. The PAA was diluted by mixing with Milli Q water in the ratio 3 to 7 (v/v). Figure 5a shows the color difference and physical appearance of the untreated and acid-treated biomass thermally-assisted pretreatment method using peracetic acid. It is to be noted the peracetic acid forms an azeotrope with water and boils at 95.5 °C at atmospheric pressure^40^. Therefore, the reaction temperature was kept at 90 °C, which is below the formation temperature of the azeotrope.

As already mentioned before, pretreatments were carried through thermally-assisted and microwave-assisted pathways. In the thermally-assisted method, wood samples and PAA mixture were poured in a round-bottom flask placed on a heating mantle (Wise Therm heating mantle, Themoline Scientific). A calibrated thermocouple was inserted into the solution to monitor and record the temperature throughout the reaction. Every thermally-assisted pretreatment run was conducted with 10 g (dry weight basis) and 200 mL of peracetic acid solution. The pretreatment temperature was fixed at 90 °C, stirring speed was 150 rpm, and reaction time was varied at 30 min, 1, 2, 3, 4, and 5 h excluding 20 min of heating time to reach the target temperature.

In the microwave-assisted pathway (Microwave synthesis reactor, Monowave 400, Anton Parr), 0.5 g of biomass, and 10 mL of peracetic acid solution was placed in a 30 mL pyrex glass tube. A PTFE septum was used to close the glass tube, and an immersion tube was inserted along with a ruby thermometer to monitor the reaction temperature. The microwave-assisted pretreatment was also conducted at 90 °C, but unlike the thermally-assisted pretreatment, the reaction time in this method was varied at 10, 20, 30 min, and 1 h. After the pretreatment, a vacuum filter was used to separate the liquid reaction mixture and the solid biomass. The moisture content of the solid sample was calculated immediately to know the solid recovery from untreated biomass using Eq. (1). After that, the recovered solid sample was thoroughly washed with MilliQ water and kept at room temperature for 72 h for air-drying.

The following equation is used to calculate the solid recovery of biomass, delignification effectiveness after pretreatment, and sugar recovery.

1 $${\text{Solid recovery}}\left( \% \right) = \frac{pretreated \, biomass \left( g \right)}{{untreated \, biomass \left( g \right)}} \times 100$$

2 $${\text{Delignification}}\left( \% \right) = \left( {{1} - \left( {\frac{{\frac{solid\;recovery\left( \% \right)}{{100}} \times lignin\;of\;pretreated\;sample\left( \% \right)}}{lignin\;of\;untreated\;biomass}} \right)} \right) \times {1}00$$

3 $${\text{Sugar}}\;{\text{recovery}}\left( \% \right) = \frac{sugar\;in\;pretreated\;biomass\left( \% \right) \times solid\;recovery\left( \% \right)}{{sugar\;in\;untreated\;biomass\left( \% \right)}}$$

### Biomass compositional analysis

Figure 5b represents a schematic of biomass pretreatment and NREL compositional analysis of the untreated and pretreated samples. A modified NREL method carried out the compositional analysis of raw and pretreated biomass^41^. The first stage was carried out at 30 °C in a water bath for an hour, where the pretreated biomass was mixed with 72% sulphuric acid. The second phase was conducted at 121 °C in a vacuum oven where the acid-biomass mixture was diluted to attain an acid concentration of 4% by adding milliQ water. In this whole process of hydrolysis, the only modification introduced was the replacement of the NREL method recommended autoclave with a vacuum oven. The solid particles retained on the frit of the glass filter are considered as acid-insoluble lignin (AIL) (Eq. 4), whereas the liquid filtrate consists of some acid-soluble lignin (ASL) and mostly sugars (carbohydrate). Sugars (glucose, xylose, mannose, arabinose, and galactose) were analysed through high-performance liquid chromatography (HPLC) (Agilent Technologies 1220 system, Alliance, USA), whereas ASL was quantified (Eq. 5) using a UV–Vis spectrophotometer (SpectraMax M2 molecular device) at a fixed wavelength of 240 nm

4 $${\text{AIL}}\left( \% \right) = \frac{residual\;lignin\left( g \right)}{{oven\;dry\;weight\;of\;sample\left( g \right)}} \times 100$$

5 $${\text{ASL}}\left( \% \right) = \frac{UVabs \times volume\;of\;filterate \times dilution}{{\varepsilon \times oven\;dry\;weight\;of\;sample \times pathlength}} \times 100$$

Here, UVabs is the UV–Vis absorbance for the sample at wavelength 240 nm, Volume of filtrate is 100 mL, Ɛ is the Absorptivity of biomass at specific 240 nm wavelength, Pathlength is the Pathlength of UV–Vis cell in cm.

The HPLC set-up for sugar analysis consisted of an Aminex HPX-87H column (300 × 7.8 mm, Bio-Rad, Hercules, USA) equipped with a refractive index detector and connected to a dual-channel gradient pump (with degasser). However, it is to be noted that this column cannot separate xylan and mannan from softwood carbohydrates since they both have the same retention time. So those two have been combined together under the section of mannan in results and discussion. For analysis, the sample injection volume was 5 µL, and all samples were analyzed in triplicates.

### Structural characterization of biomass

The structure of untreated and pretreated biomass was characterized by analyzing crystallinity, surface morphology and change of functional groups.

The crystallinity of untreated and pretreated biomass was determined using an X-ray diffractometer (Rigaku Miniflex600, Detector: D/teX Ultra with florescent X-ray reduction mode option) equipped with a Cu radiation. The biomass was scanned in the range of 10°–30° at a rate of 0.005° per second. The crystallinity index (*CrI*) was analyzed according to the Segal method based on the peak height by using the following equation^42^

6 $$CrI = \frac{{I_{002} - I_{am} }}{{I_{002} }} \times 100$$

here *I*_*002*_ is the maximum scattered intensity of the crystalline portion at 22°, and *I*_*am*_ is the minimum intensity of the amorphous region at 18° which is considered as the secondary peak.

Surface morphology of untreated biomass and their changes after pretreatment were identified through scanning electron microscopy (SEM) (Phenom XL Desktop SEM, Thermo Scientific, USA) at an accelerating voltage of 5 kV or 10 kV. Before the SEM analysis, gold coating was used by a sputter to improve the conductivity of the sample surface because biomass samples are non-conductive.

To investigate the chemical structure and functional group changes in biomass, Fourier transform infrared (FTIR) spectroscopy was conducted using a (Perkin Elmer Spectrum FT-IR Spectrometer, Universal ATR with diamond/ZnSe crystal. The deformation of the raw material and pretreated biomass occurred due to the stretching of bonds in cellulose, hemicellulose, and lignin. The untreated and pretreated biomass were placed on the diamond crystal, and a torque knob was used for applying the same pressure for all measurements. The FTIR spectra were recorded between the mid-IR range of 4000–450 cm^−1^ with 32 scans at a resolution of 4 cm^−1^. Checks on contamination of the diamond crystal surface and background scanning were carried out for every sample.

The elemental composition of untreated and pretreated biomass was determined using Thermo scientific flashmart CHNS analyser. The reactor temperature was fixed at 950 °C. The carrier gas was helium at 140 mL min^−1^, and the detector was TCD (with reference helium at 100 mL min^−1^). The standard we used is BBOT, and the run time was 12 min. Oxygen was injected at 250 mL min^−1^ flow rate for 5 s during the analysis.

## Conclusions

This study demonstrated an effective and reproducible method of biomass delignification by peracetic acid resulting in the maximum digestion of biomass. The thermally-assisted pretreatment at 90 °C for 5 h effectively removed both hemicellulosic sugar (xylan and mannan) and lignin from both hardwood and softwood. Softwood delignification rate is slower compared to the hardwood. Both the biomass obtained high glucan recovery after delignification. Also, this pretreatment method enables low-energy operation to maximize the production of glucan rich feedstock supply chain. This thermally-assisted pretreatment under mild condition is a promising alternative method for the efficient lignin removal from lignocellulosic biomass with enriching of glucan content for further processing. Our ongoing work involves scaling-up the process and use of this pretreated biomass for LGO and 5-CMF production.

## Supplementary Information

Supplementary Information.
