# Supplementary material for: One-step peracetic acid pretreatment of hardwood and softwood biomass for platform chemicals production
Source: Sci Rep. 2021 May 27;11:11183. doi: 10.1038/s41598-021-90667-9 (PMC8160206; doi:10.1038/s41598-021-90667-9)
Supplement: Supplementary file 1 — Supplementary Information. [file 41598_2021_90667_MOESM1_ESM.docx]

**Supplementary information**

Supplementary Table S1. Effect of microwave-assisted pretreatment conditions on the hardwood biomass compositions, the recovery of solid and carbohydrates.

|  | **Carbohydrate (%)** | | | |  |  |  |  |  | **Carbohydrate recovery (%)** | | | |
| --- | --- | --- | --- | --- | --- | --- | --- | --- | --- | --- | --- | --- | --- |
| **Time** | **Glucan %** | **Xylan %** | **Arabinan %** | **Galactan %** | **Lignin %** | **AIL %** | **ASL %** | **Delignification %** | **Solid recover (%)** | **Glucan recovery%** | **Xylan recovery %** | **Arabinan recovery** | **Galactan recovery (%)** |
| **Raw-Hardwood** | 40.51 | 22.12 | 1.06 | ND | 32.24 | 29.65 | 2.59 |  |  |  |  |  |  |
| **Hardwood**  **10 min** | 48.19 | 17.00 | 0.78 | ND | 21.22 | 18.83 | 2.39 | 51.08 | 79.46 | 94.53 | 61.07 | 58.47 | ND |
| **Hardwood**  **20 min** | 51.73 | 13.66 | 0.77 | ND | 18.70 | 16.43 | 2.27 | 59.83 | 74.77 | 95.48 | 46.17 | 73.81 | ND |
| **Hardwood**  **30 min** | 53.43 | 13.50 | 0.66 | ND | 17.17 | 15.01 | 2.16 | 64.89 | 71.54 | 94.36 | 43.66 | 61.32 | ND |
| **Hardwood 1 hr** | 57.28 | 11.69 | 0.69 | ND | 12.97 | 10.97 | 2.00 | 75.66 | 67.84 | 95.92 | 35.85 | 31.72 | ND |

Supplementary Table S2. Effect of microwave-assisted pretreatment conditions on the softwood biomass compositions, the recovery of solid and carbohydrates.

|  | **Carbohydrate (%)** | | | |  |  |  |  | |  | **Carbohydrate recovery (%)** | | | |
| --- | --- | --- | --- | --- | --- | --- | --- | --- | --- | --- | --- | --- | --- | --- |
| **Time** | **Glucan (%)** | **Xylan (%)** | **Arabinan (%)** | **Galactan (%)** | **Lignin (%)** | **AIL (%)** | **ASL (%)** | **Delignification (%)** | | **Solid recover (%)** | **Glucan (%)** | **Mannan (%)** | **Arabinan (%)** | **Galactan (%)** |
| **Raw-Softwood** | 39.62 | 21.77 | 1.31 | 0.36 | 36.70 | 34.27 | 2.43 |  |  | |  |  |  |  |
| **Softwood 10 min** | 43.88 | 16.87 | 1.50 | 0.34 | 33.95 | 31.71 | 2.24 | 26.72 | 85.81 | | 96.04 | 65.45 | 98.26 | 81.05 |
| **Softwood 20 min** | 45.80 | 15.31 | 1.44 | 0.30 | 31.57 | 29.28 | 2.29 | 34.94 | 82.50 | | 95.37 | 57.10 | 79.20 | 68.75 |
| **Softwood 30 min** | 47.42 | 13.99 | 1.26 | 0.26 | 27.84 | 25.69 | 2.15 | 44.06 | 80.86 | | 96.77 | 51.14 | 70.75 | 58.40 |
| **Softwood 1 hr** | 49.13 | 12.55 | 1.09 | 0.20 | 23.14 | 22.60 | 2.01 | 51.67 | 77.55 | | 96.17 | 44.00 | 58.70 | 43.08 |

Supplementary Fig. S1. The recovered hardwood and softwood biomass based on the content of each component in 100 g of biomass before and after the pretreatment.
